# The Consumption of Non-Sugar Sweetened and Ready-to-Drink Beverages as Emerging Types of Beverages in Shanghai

**DOI:** 10.3390/nu16203547

**Published:** 2024-10-19

**Authors:** Zhengyuan Wang, Liping Shen, Jinpeng Ning, Zhuo Sun, Yiwen Xu, Zehuan Shi, Qi Song, Wei Lu, Wenqing Ma, Shupeng Mai, Jiajie Zang

**Affiliations:** 1 Department of Nutrition and Health, Division of Health Risk Factors Monitoring and Control, Shanghai Municipal Center for Disease Control and Prevention, Shanghai 200336, China; wangzhengyuan@scdc.sh.cn (Z.W.); shenliping@scdc.sh.cn (L.S.); sunzhuo@scdc.sh.cn (Z.S.); shizehuan@scdc.sh.cn (Z.S.); songqi@scdc.sh.cn (Q.S.); luwei@scdc.sh.cn (W.L.); mawenqing@scdc.sh.cn (W.M.); maishupeng@scdc.sh.cn (S.M.); 2 School of Public Health, Shanghai University of Traditional Chinese Medicine, Shanghai 201203, China; 17505564961@163.com; 3 The College of Medical Technology, Shanghai University of Medicine and Health Sciences, Shanghai 201318, China; xu_yw1021@163.com

**Keywords:** beverage, non-sugar sweetened beverages, ready-to-drink beverages, retail visitors, Shanghai

## Abstract

Background: The Chinese beverage industry is experiencing rapid growth, particularly in the popularity of non-sugar sweetened beverages (NSSs) and ready-to-drink beverages (RSBs). This study aimed to assess current consumption patterns and determinants of various beverage types among retail visitors. Methods: A total of 44 observation points, including 22 supermarkets and 22 convenience stores, were randomly selected across Shanghai. At each location, at least 100 individuals were recruited to participate. Data were collected using an electronic self-administered questionnaire. Results: The consumption rates of total beverages, sugar-sweetened beverages, NSSs, and RSBs were 57.70%, 56.94%, 19.60%, and 29.50%, respectively; the median consumption amounts among the drinking population were 162.57 mL/day, 137.98 mL/day, 32.85 mL/day, and 32.85 mL/day, respectively. The consumption proportions of NSSs and RSBs ranked 2nd and 3rd. The multifactorial analyses showed that people aged 6–18 years consumed more beverages (*p* < 0.05). Males were more likely to consume sugar-sweetened beverages and NSSs, but females were more likely to consume RSBs (*p* < 0.05). Higher educated people and bachelors were more likely to consume beverages (*p* < 0.05). Conclusions: The emerging beverage categories, NSSs and RSBs, warrant attention due to their significant consumption rates. Tailored intervention strategies should be considered for demographic groups varying by age, gender, and educational attainment.

## 1. Introduction

In recent years, the global beverage industry has experienced rapid expansion, with a corresponding surge in beverage consumption. According to data from the National Bureau of Statistics [1], China’s beverage industry output reached 175 million tons in 2023, marking a staggering 37-fold increase over the past three decades, with an impressive average annual growth rate of 13%. Per capita beverage consumption has soared to 30 times its level of 30 years ago, a growth trajectory that merits significant attention. Sugar-sweetened beverages (SSBs), favored for their diversity and convenience, dominate the market, with a notable uptick in consumption. A global analysis of SSB intake among adults across 185 countries revealed a 91.76 g/week increase from 1990 to 2018, with sub-Saharan Africa witnessing the most substantial rise of 741.52 g/week [2]. While China’s SSB consumption does not lead globally, the upward trend is unmistakable [2]. A 2016 survey across 27 Chinese cities indicated that a substantial majority—74% of children (4–9 years), 85% of adolescents (10–17 years), and 83% of adults (18–55 years)—consumed at least 500 mL of SSBs weekly [3].

An umbrella review [4] had shown that high dietary sugar consumption was associated with a range of health risks. SSBs are the largest source of dietary sugars, so the intake of SSBs has become an urgent public health problem. Studies have shown that for every additional 355 mL of SSB consumed per day, the body weight of adults would increase by 0.22 kg and the body mass index of children would increase by 0.06 kg/m^2^ in 1 year [5]. To add insult to injury, SSBs are also associated with an increased risk of cardiovascular disease. A meta-analysis of seven prospective cohort studies [6] found that consuming SSBs one or more times per day increased the risk of cardiovascular disease by approximately 9% compared with consuming no SSBs or less than one SSB per month.

In addition to various chronic diseases, SSBs increase the risk of death. A 2010 analysis of data from the Global Dietary Surveys estimated that 184,000 people die each year due to excessive intake of SSBs [7]. The Nurses’ Health Study and Health Professionals Follow-up Study also found [8] that there was a positive association between SSBs and mortality, which was mainly correlated with mortality through cardiovascular disease. This implies that long-term excessive SSB intake may increase the risk of mortality.

High SSB consumption is linked to a host of health issues. Consequently, many have switched to non-sugar sweetened options to indulge their sweet tooth without the caloric intake. In China, NSS sales hit $0.21 billion in 2019, growing at a CAGR of 42.84%, and are projected to reach $0.57 billion by 2027 [9]. The WHO guidelines flag potential NSS health risks such as cardiovascular disease and metabolic issues, calling for more research on their safety and health impact. They also note that NSS benefits generally exceed the drawbacks, underscoring the importance of mindful NSS consumption [10]. On the other hand, beyond traditionally packaged SSBs, the market for ready-to-drink beverages (RSBs) is burgeoning in China. Data from China Report Hall [11] indicates that China’s RSB sales surpassed $14.36 billion in 2022, with projections suggesting a leap to over $28.72 billion by 2025, reflecting a swift growth trajectory. Prior research on beverage consumption has predominantly centered on packaged SSBs, with scant attention to RSBs, potentially leading to an underestimation of overall beverage intake. Consequently, our study aimed to assess the current landscape of beverage consumption alongside potential influencing factors among Shanghai’s residents in 2023.

## 2. Method

### 2.1. Participants

In this study, we utilized the shopping mall intercept methodology commonly recognized in market research to randomly engage visitors at the retail location for our survey, taking into account both those who made purchases and those who did not, thereby providing a comprehensive dataset for analysis. This study first randomly selected eight districts with different economic levels and geographical locations (namely Jiading, Pudong, Putuo, Changning, Xuhui, Jing’an, Songjiang, Fengxian) from the 16 districts in Shanghai. Then, 22 supermarkets and 22 convenience stores were randomly selected as observation points within these selected districts. For each observation point, at least 40 people were randomly recruited from the age group of 6–18 years old, and at least 20 people were randomly recruited from each of the age groups of 18–40 years old, 40–60 years old, and >60 years old, with an equal distribution between gender with each age group.

### 2.2. Data Collection

Our survey was conducted from June to July in 2023. We used a self-administered electronic questionnaire, which took approximately 8–10 min to complete. The uniformly trained surveyors guided and assisted the participants in filling out the questionnaires after obtaining informed consent. Demographic information (e.g., gender, age, personal income, etc.), Noncommunicable Chronic Disease status (NCD), height, weight, and beverages consumption status were collected through the questionnaire. The beverages included nine types such as NSSs, carbonated beverages (CBs), fruit-flavored beverages (FFBs, including 100% NFC), sugar-sweetened tea beverages (STBs), vegetable protein beverages (VPBs), lactic acid bacteria beverages (LABs), RSBs, sports functional beverages (SFBs), and sugar-sweetened coffee beverages (SCBs). The term “beverages” did not include water, and no information related to it was surveyed. The participants were asked about the consumption frequency and average amount of each type of beverage in the past month. The consumption frequency was divided into five groups: no drink, 1–3 times/month, 1–3 times/week, 4–7 times/week, and >1 time/day. We showed the survey participants the common sizes of bottled beverages to help them estimate their average consumption.

### 2.3. Covariates and Categorization

The education level was classified into three groups: low education (junior high school and below), medium education (senior high school/secondary school/technical school/college), and high education (bachelor’s degree and above). Marriage status analysis did not include people aged 6–18 years. Personal income, after excluding those aged 6–18 years old, was classified into low income (<$500/month), medium income ($500–1000/month), and high income (>$1000/month). Obesity was defined according to the standards of the National Health and Wellness Commission of the People’s Republic of China [12,13,14]. We divided the beverages into two categories: SSBs and NSSs, based on whether they contain added sugar. The SSBs included CBs, FFBs, STBs, VPBs, LABs, RSBs, SFBs, and SCBs. Total beverages (TBs) equal NSSs plus SSBs. The beverage consumption population was defined as those who had drank beverages at least once in the past month.

### 2.4. Statistical Analysis

Statistical analyses were completed with SPSS 25.0. All statistical significance tests were two-sided, and the level of *p* < 0.05 was considered statistically significant. The chi-square test was used to analyze qualitative variables, and quantitative variables were compared using non-parametric tests. The binary logistic regression analyses were used to analyze the factors influencing the intake of beverages.

## 3. Results

### 3.1. Basic Information of the Survey Population

The total number of respondents for this study was 6549. After excluding the failed questionnaires, the final sample consisted of 6394 respondents, with a pass rate of 98.0%. Of these, 3135 (49.03%) were males and 3259 (50.97%) were females, with an average age of 35.8 ± 21.79 years. There were significant statistical differences in the composition ratios for personal income, occupational status, marriage status, and obesity between males and females (*p* < 0.05), as shown in Table 1. The composition ratios of other survey factors did not show statistically significant differences.

### 3.2. Consumption Rate and Consumption Amount of Different Types of Beverages

The consumption rates of TBs, SSBs, NSSs, and RSBs were 57.70%, 56.94%, 19.60%, and 29.90%, respectively, in Table 2. The consumption rates of the four types of beverages among different age, personal income, education level, marriage status, NCD, and weight loss/shaping groups showed statistically significant differences (*p* < 0.05). In addition to these variables, the four types of beverages had their own characteristics. There were significant statistical differences in the consumption rates of NSSs and RSBs across the gender index, in the consumption rates of TBs, SSBs, and NSSs across the occupational status and obesity indices, and in the consumption rates of SSBs and NSSs across the mood swing index (*p* < 0.05).

The median consumption amounts of TBs, SSBs, NSSs, and RSBs in the drinking population ware 162.57 mL/day, 137.98 mL/day, 32.85 mL/day, and 32.85 mL/day, respectively, in Table 2. There were significant statistical differences in the consumption amounts of TBs and SSBs among different gender, age, personal income, education level, occupational status, marriage status, obesity, as well as weight loss/shaping and mood swing groups (*p* < 0.05). Moreover, there were significant statistical differences in the consumption amount of SSBs across the NCD index, in the consumption amounts of NSSs across the gender, occupational status, NCD, obesity, and mood swing indices, and in the consumption amounts of RSBs across the age, occupational status, and marriage status, NCD, obesity, weight loss/shaping and mood swing indices (*p* < 0.05).

### 3.3. Consumption Amount and Frequency Distribution of Beverages

**Figure 1:**
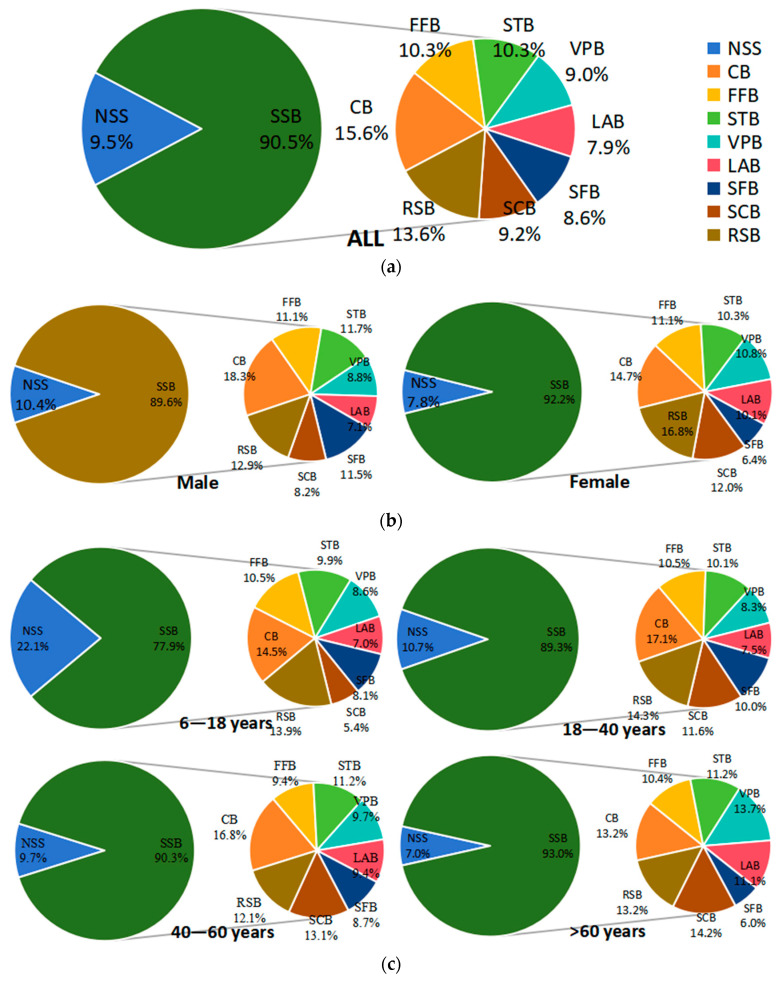

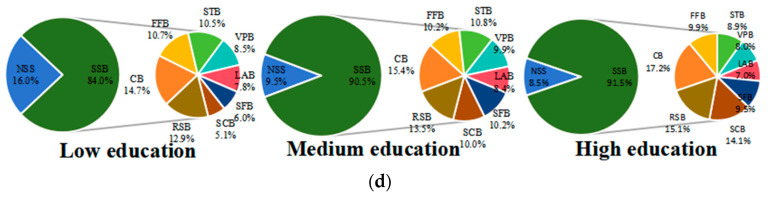
The consumption amount distribution of various types of beverages; (**a**–**d**), respectively, display the composition ratios of various beverage consumptions for all subjects, different genders, different age groups, and different levels of education. (NSSs = non-sugar sweetened beverages; SSBs = Sugar-sweetened beverages; CBs = carbonated beverages; FFBs = fruit-flavored beverages (including 100% NFC); STBs = sugar-sweetened tea beverages; VPBs = vegetable protein beverages; LABs = lactic acid bacteria beverages; SFBs = sports functional beverages; SCBs = sugar-sweetened coffee beverages; RSBs = ready-to-drink beverages).

**Figure 2:**
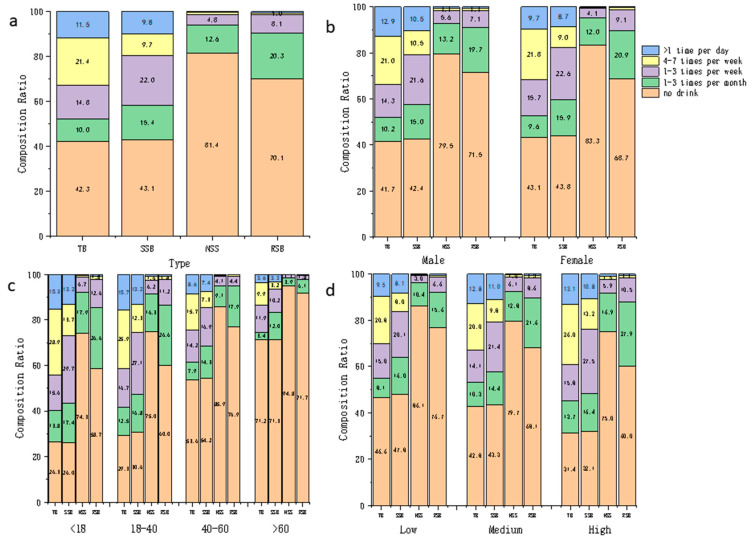
Consumption frequency distribution of beverages (%); (**a**–**d**), respectively, demonstrate the composition ratios of different consumption frequencies for four types of beverages in all subjects, different genders, different age groups, and different levels of education. (Total = total beverages; SSBs = sugar-sweetened beverages; NSSs = non-sugar sweetened beverages; RSBs = ready-to-drink beverages).

Of TB consumption amount, SSB consumption accounted for 84.5% and NSS consumption accounted for 9.5%. The largest share of SSBs was CBs with 15.6% and the smallest was LABs with 7.9%. RSBs had a slightly lower share than CBs, coming in second with 13.6%, in Figure 1a. Moreover, the consumption amount distribution of various types of beverages across different genders, ages, and education level groups are shown in Figure 2b–d.

The proportion of people who drank TBs, SSBs, NSSs, and RSBs at least once a week were 47.7%, 41.6%, 6.0%, and 9.6%, respectively, in Figure 1a. Moreover, the consumption frequency distribution of various types of beverages across different genders, ages, and education level groups are shown in Figure 2b–d.

### 3.4. The Multifactorial Analyses Result of Influencing Factors Related to the Consumption of Beverages

Logistic regression analysis was conducted with gender, age, education level, occupational status, marriage status, obesity, NCD, weight loss/shaping, and mood swing as independent variables, and the consumption rate of different types of beverages as dependent variables in Table 3.

Logistic regression analysis of TBs showed that, compared with the 6–18 age group, the risk decreased in the 18–40 age group (OR = 0.585, 95% CI: 0.476–0.720); the 40–60 age group (OR = 0.310, 95% CI: 0.241–0.400), and the >60 age group (OR = 0.191, 95% CI: 0.147–0.249). Compared with the low-education group, the risk increased by 49.0% (OR = 1.490, 95% CI: 1.306–1.700) in the middle-education group and 118% (OR = 2.180, 95% CI: 1.809–2.626) in the high-education group. Compared with the married group, the risk increased in the bachelor group (OR = 1.551, 95% CI: 1.266–1.899). Those with weight loss/shaping had a 70.4% increased risk (OR = 1.704, 95% CI: 1.375–2.111).

Similar results were found in the analyses of the other three types of beverages in the different age, education level, marriage status, and weight loss/shaping groups. In addition to these variables, the other three types of beverages had their own characteristics. Females had a lower risk of consuming NSSs (OR = 0.781, 95% CI: 0.684–0.892) but a higher risk of consuming RSBs (OR = 1.197, 95% CI: 1.067–1.343) than males. People working in health-related industries were at higher risk of consuming TBs and SSBs [(OR = 1.298, 95% CI: 1.127–1.496); OR = 1.257, 95% CI: 1.092–1.446)].

## 4. Discussion

In recent years, global production and consumption of beverages have been on the rise. At the same time, numerous studies [15,16] have shown that excessive intake of SSBs and NSSs can cause various health problems. According to our study, the consumption rates of TBs, SSBs, NSSs, and RSBs in Shanghai were 57.70%, 56.94%, 19.60%, and 29.50%, respectively. The 2017–2018 U.S. National Health and Nutrition Examination Survey (NHANES) [17] showed that the percentage of adults aged 20–39 years consuming any amount of SSBs on a given day was 72.6% across all races and/or ethnicity groups. A national survey of Australians [18] aged 18 years and older showed that almost half (47.3%) of participants had consumed an SSB in the past week, with 13.6% consuming it at least once a day. In our survey, about 34.3% of participants aged 18 years and older had consumed an SSB in the past week, with 8.1% consuming it at least once a day on average. Although the SSB consumption level in Shanghai is lower than in developed countries such as the United States and Australia, the sales data of two major brands, the Coca-Cola Company (Atlanta, GA, USA) and PepsiCo Inc. (New York, NY, USA). [19], and studies [20,21], all showed that the consumption of SSBs in China is on a growing trend. The rate of SSB consumption at least once every 3 days among urban residents was 11.4% in 18 provinces of China, and the average SSB consumption amount in the drinking population was 130.0 g/d [20]. Corresponding to our survey, the rate of SSB consumption at least 4 times/week was 19.5%, nearly twice the national level, and the average consumption in the drinking population was 412.0 mL/day, which is over three times the national level. The high consumption rate of beverages in Shanghai may be related to the fact that the level of economic development in Shanghai is among the highest in the country. The article about global nutrition dynamics published by the American Journal of Clinical Nutrition [22] showed that an increase in the level of economic development may bring about changes in the eating habits and lifestyles of the residents, such as purchasing more SSBs, etc.

Regarding TB consumption, CBs still hold the largest share. Additionally, emerging types of beverages were also very much worth noting. RSBs and NSSs accounted for 12.3% and 9.5%, respectively, ranking them second and third. They have become the main types of beverages, and a lot of analyses [9,10] showed that the consumption proportion in Chinese consumers is still expanding. RSB ingredients are opaque, and there is a misconception about NSSs that many people think they are healthier than pre-packaged SSBs because of their low-calorie characteristic. Therefore, it is necessary to guide people to recognize and learn about new types of beverages and strengthen health education in them.

In our study, both consumption rate and consumption amount showed a downward trend with increasing age. In a systematic assessment of beverage intake in 187 countries/regions worldwide, it was shown that the consumption of SSBs usually followed an inverse age gradient [23], a finding that was also confirmed in our study. The NHANES showed that children (aged 2 to 19) always consume SSBs at a higher rate than adults (aged 20+) [24]. A study investigating the consumption of SSBs in São Paulo found that adolescents had the highest consumption of SSBs [25]. These data showed that adolescents are the main consumers of beverages. The excessive consumption of SSBs poses a threat to the healthy development of children and adolescents, especially by increasing the risk of obesity [6,26] and dental caries [15,16]. Therefore, for the adolescent population, we need to strengthen guidance and regulation of beverage consumption to control their intake of SSBs.

There were differences in beverage consumption preferences between males and females. Our study found that although the difference in SSB consumption rates between males and females was not statistically significant (*p* = 0.192), the consumption amount was higher among males than females, which is consistent with the results for Chinese children and adolescents [26] and for the U.S. in all age groups [16]. Similar results had been found in São Paulo, Taiwan, and several other regions around the world [23,25,27]. The reasons females consume less SSBs may include a lower energy intake requirement resulting in a lower total food intake, and that females have growing concern for their appearances and increasing health awareness compared with males [28]. In addition, this study also found that the consumption rate of RSBs in females was higher than in males. The Report of Development Prospect Prediction and Investment Strategy Planning on China New Tea Drinking Industry (2023–2028) described that women’s consumption rate of RSBs was higher than men’s, with women’s share reaching 65.09% in 2022 [29]. These data further proved the difference in beverage consumption preferences between different gender groups.

The beverage consumption was also closely related to economic level. When analyzing the association between income and educational level with the beverage consumption, we found that the highest consumption rates and amounts of TBs, SSBs, NSSs, and RSBs were in the groups with the highest incomes and the highest educational level. This is similar to some other national studies. In Mexico, two nationally representative dietary intake surveys reported higher average energy intake of SSBs for children from higher socioeconomic status families and from urban families compared with rural families [30]. The Korean National Health and Nutrition Examination Survey showed that people in the highest income group were more likely to consume SSBs (OR = 1.18 for females and 1.25 for males) [31]. However, there are also some inconsistent studies. The result of a survey on the consumption patterns of SSBs in the United States showed that lower-income and lower educational attainment people consumed more SSBs [32]. Australia also found higher odds of SSB consumption and higher caloric intake among people of low income [33]. This research inconsistency may be related to the development of economic levels in different countries. People with higher incomes in economically developed countries have additional nutritional knowledge and preventive information about nutrition and tend to choose healthy foods [34], whereas in countries with relatively weaker economies, people with low incomes decline purchasing higher-price foods. China is still a developing country, but the price of beverages is often relatively high, for example, a cup of RSB costs about $2–4. This may be the reason why people with low incomes tend not to consume beverages. It suggests that we also should strengthen the health education of high-income people to raise awareness of the potential negative effects of beverages consumption.

We also found an interesting phenomenon: the bachelor consumed more beverages than married person. This phenomenon may be due to the fact that there is a positive relationship between being married and being healthy [35,36], as partners are bound to each other, which helps to form healthy habits, and married persons may consume healthier foods and fewer beverages on a daily basis. Research on the relationship between marriage and health showed that emerging adults with an unhealthy BMI have an improved BMI after entering marriage, further favoring chronic disease management and improving future health [37,38,39]. This is similar to our findings.

In our study, we delved into the beverage consumption patterns across various demographic groups in Shanghai. Our comprehensive inclusion of different beverage types, especially emerging types of beverages such as NSSs and RSBs, which were the beverage categories that have rarely been studied separately in the past but have increasing significant consumption, coupled with a rational sampling strategy and a robust sample size, has effectively mitigated the shortcomings observed in prior research. This has allowed us to present more representative insights into the consumption of beverages in the region.

However, there are some limitations in our study. While our work provided a temporal snapshot and reduced recall bias, it may not fully capture the complexities of long-term beverage consumption habits. Our survey focused on retail visitors, which may limit the generalizability of our findings to the broader population. Additionally, household income indeed may affect the beverage consumption of kids, but our survey did not cover this aspect.

## 5. Conclusions

In summary, the beverage consumption in Shanghai, although lower than internationally, is still high and suggests that timely interventions are needed. Firstly, we need to strengthen health education, especially for adolescents, who are the main consumers of SSBs. Secondly, health education should not only focus on CBs, which were the most significant contributors to TB consumption, but also pay attention to emerging types of beverages, such as NSSs and RSBs. Thirdly, there should be different intervention strategies for different genders, e.g., males should focus on controlling SSB and NSS intake, while females should focus on preventing excessive RSB intake. Finally, health awareness needs to be raised even among high-income and highly educated populations.

## Figures and Tables

**Table 1:** Basic information of the survey population.

	Male	Female	Total	χ^2^ ^(1)^	*p* ^(2)^
Total	3135 (49.03%)	3259 (50.97%)	6394 (100%)		
Age					
6–18	1053 (33.59%)	1034 (31.73%)	2087 (32.64%)	3.987	0.263
18–40	748 (23.86%)	770 (23.63%)	1518 (23.74%)		
40–60	670 (21.37%)	753 (23.11%)	1423 (22.26%)		
>60	664 (21.18%)	702 (21.54%)	1366 (21.36%)		
Personal income					
low	458 (22.00%)	610 (27.42%)	1068 (24.80%)	52.02	<0.001
medium	942 (45.24%)	1099 (49.39%)	2041 (47.39%)		
high	682 (32.76%)	516 (23.19%)	1198 (27.82%)		
Education level					
low	1263 (40.29%)	1344 (41.24%)	2607 (40.77%)	4.67	0.099
medium	1310 (41.79%)	1282 (39.34%)	2592 (40.54%)		
high	562 (17.93%)	633 (19.42%)	1195 (18.690%)		
Occupational status					
health-related industries	607 (19.36%)	710 (21.79%)	1317 (20.60%)	5.74	0.017
others	2528 (80.64%)	2549 (78.21%)	5077 (79.40%)		
Marriage status					
bachelor	1502 (47.91%)	1423 (43.67%)	2925 (45.75%)	16.372	<0.001
married	1569 (50.05%)	1735 (53.24%)	3304 (51.67%)		
divorced/widowed	64 (2.04%)	101 (3.10%)	165 (2.58%)		
Obesity					
yes	443 (14.13%)	325 (9.97%)	768 (12.01%)	26.15	<0.001
no	2692 (85.87%)	2934 (90.03%)	5626 (88.99%)		
NCD ^(3)^					
yes	633 (20.19%)	602 (18.47%)	1235 (19.31%)	3.03	0.082
no	2502 (79.81%)	2657 (81.53%)	5159 (80.69%)		
Weight loss/shaping in the past month					
yes	249 (7.94%)	299 (9.18%)	548 (8.57%)	3.10	0.079
no	2886 (92.06%)	2960 (90.83%)	5846 (91.43%)		
Mood swing in the past month					
yes	72 (2.30%)	81 (2.49%)	153 (2.39%)	0.24	0.621
no	3063 (97.70%)	3178 (97.52%)	6241 (97.61%)		

^(1)^ Chi-square test statistics; ^(2)^ significance of the test, considering a level of 5%; ^(3)^ NCD = Noncommunicable Chronic Diseases.

**Table 2:** Consumption rate and consumption amounts of beverages.

Variables	TB ^(1)^				SSB ^(2)^				NSS ^(3)^				RSB ^(4)^			
	Drink Rate (%)	*p* ^(5)^	Consumption Amount Medium (P25, P75)	*p*	Drink Rate (%)	*p*	Consumption Amount Medium (P25, P75)	*p*	Drink Rate (%)	*p*	Consumption Amount Medium (P25, P75)	*p*	Drink Rate (%)	*p*	Consumption Amount Medium (P25, P75)	*p*
Gender		0.23		<0.001		0.192		<0.001		<0.001		0.001		0.015		0.940
male	58.30		175.71 (65.70, 396.56)		57.80		160.97 (65.70, 359.83)		20.50		32.85 (32.85, 85.71)		28.50		32.85 (32.85, 142.86)	
female	56.90		142.86 (52.56, 302.14)		56.20		133.38 (49.28, 285.72)		16.70		32.85 (21.68, 85.71)		31.30		32.85 (32.85, 142.86)	
Age		<0.001		<0.001		<0.001		<0.001		<0.001		0.056		<0.001		0.001
6–18	73.70		174.11 (65.70, 379.98)		75.80		131.41 (39.42, 292.10)		28.20		32.85 (32.85, 142.86)		39.30		32.85 (32.85, 142.86)	
18–40	70.70		176.14 (65.70, 384.27)		69.40		165.85 (65.70, 357.14)		25.00		32.85 (32.85, 142.86)		40.00		32.85 (32.85, 142.86)	
40–60	46.40		142.86 (45.99, 357.14)		45.80		132.85 (45.99, 320.21)		14.10		32.85 (32.85, 142.86)		23.10		32.85 (32.85, 65.70)	
>60	28.80		77.43 (32.85, 215.13)		28.70		72.92 (32.85, 214.29)		5.20		32.85 (19.71, 96.42)		8.30		32.85 (21.68, 131.41)	
Personal income		<0.001		<0.001		<0.001		<0.001		<0.001		0.568		<0.001		0.518
low	40.20		134.00 (42.86, 301.42)		39.50		129.99 (45.01, 282.85)		11.00		32.85 (23.00, 94.29)		17.50		32.85 (32.85, 137.13)	
medium	45.50		134.00 (49.28, 318.57)		44.90		128.27 (45.99, 289.89)		12.60		32.85 (32.85, 131.41)		22.10		32.85 (32.85, 98.55)	
high	64.20		178.57 (65.70, 419.28)		63.40		171.43 (65.70, 374.46)		23.00		32.85 (32.85, 142.86)		34.30		32.85 (32.85, 142.86)	
Education level		<0.001		<0.001		<0.001		<0.001		<0.001		0.094		<0.001		0.374
low	53.40		131.41 (49.28, 294.24)		53.80		101.99 (32.85, 241.77)		15.90		32.85 (21.68, 142.86)		22.80		32.85 (32.85, 142.86)	
medium	57.20		177.40 (65.70, 416.55)		57.70		156.00 (54.53, 357.14)		20.90		32.85 (32.85, 142.86)		31.50		32.85 (32.85, 142.86)	
high	68.60		175.71 (65.70, 381.51)		68.00		164.26 (65.70, 334.10)		25.00		32.85 (26.28, 142.86)		40.00		32.85 (32.85, 131.41)	
Occupational status		<0.001		0.004		<0.001		<0.001		<0.001		<0.001		0.102		0.003
health-related industries	63.20		175.71 (65.70, 423.21)		66.10		98.55 (32.85, 274.26)		25.90		65.70 (32.85, 228.57)		31.20		32.85 (29.07, 122.85)	
others	56.10		154.40 (57.14, 342.14)		55.60		142.86 (54.53, 314.98)		17.80		32.85 (26.28, 100.00)		29.00		32.85 (32.85, 142.86)	
Marriage status		<0.001		<0.001		<0.001		<0.001		<0.001		0.324		<0.001		<0.001
bachelor	74.70		175.71 (65.70, 366.06)		73.70		161.54 (65.70, 339.20)		26.40		32.85 (29.57, 131.41)		42.40		32.85 (32.85, 142.86)	
married	43.40		131.41 (41.71, 307.11)		42.80		118.27 (39.42, 285.71)		12.00		32.85 (26.28, 98.55)		19.30		32.85 (32.85, 98.55)	
divorced/widowed	45.20		146.99 (46.98, 368.42)		44.60		142.86 (44.02, 282.85)		12.50		52.56 (21.68, 142.86)		19.60		32.85 (26.28, 142.86)	
Obesity		0.002		<0.001		0.003		0.001		0.001		0.004		0.123		0.003
yes	62.80		177.40 (65.70, 463.11)		62.00		165.06 (65.70, 425.70)		22.80		32.85 (32.85, 142.86)		32.30		39.42 (32.85, 142.86)	
no	56.90		151.76 (55.85, 328.68)		56.30		142.86 (52.56, 305.43)		18.00		32.85 (24.97, 98.55)		29.60		32.85 (32.85, 142.86)	
NCD ^(6)^		<0.001		0.850		<0.001		<0.001		<0.001		<0.001		<0.001		0.014
yes	41.00		161.10 (52.56, 405.67)		43.90		65.70 (13.14, 236.81)		15.30		124.84 (32.85, 357.14)		16.90		32.85 (26.28, 98.55)	
no	62.10		162.57 (61.42, 354.25)		61.70		142.86 (55.85, 318.57)		20.70		32.85 (27.43, 131.41)		32.80		32.85 (32.85, 142.86)	
Weight loss/shaping in the past month		<0.001		<0.001		<0.001		<0.001		<0.001		0.055		<0.001		0.034
yes	74.30		220.71 (98.55, 485.69)		73.50		197.71 (91.33, 423.69)		32.80		32.85 (32.85, 142.86)		46.90		32.85 (32.85, 142.86)	
no	56.20		151.12 (55.85, 342.86)		56.50		131.41 (39.42, 289.75)		18.40		32.85 (29.07, 142.86)		27.90		32.85 (32.85, 142.86)	
Mood swing in the past month		0.097		<0.001		<0.001		<0.001		<0.001		<0.001		0.117		<0.001
yes	62.30		295.66 (127.14, 571.43)		82.00		1.85 (0, 175.71)		37.30		274.26 (65.70, 714.29)		25.40		32.85 (19.71, 45.99)	
no	57.30		153.09 (57.14, 342.86)		56.70		142.86 (54.53, 318.57)		18.50		32.85 (26.28, 107.14)		30.70		32.85 (32.85, 142.86)	
All	57.70		162.57 (59.13, 357.14)		56.94		137.98 (45.99, 306.06)		19.60		32.85 (32.85, 142.86)		29.90		32.85 (32.85, 142.86)	

^(1)^ Total beverages; ^(2)^ sugar-sweetened beverages; ^(3)^ non-sugar sweetened beverages; ^(4)^ ready-to-drink beverages; ^(5)^ significance of the test, considering a level of 5%; ^(6)^ NCD = Noncommunicable Chronic Diseases.

**Table 3:** Logistic regression analyses of influencing factors related to the consumption of beverages.

	TB ^(1)^				SSB ^(2)^				NSS ^(3)^				RSB ^(4)^			
	β ^(5)^	*p*	OR ^(5)^	95% CI ^(5)^	β	*p*	OR	95% CI	β	*p*	OR	95% CI	β	*p*	OR	95% CI
Gender																
male	reference				reference				reference				reference			
female	−0.037	0.507	0.964	0.864–1.075	−0.043	0.432	0.957	0.859–1.067	−0.247	<0.001	0.781	0.684–0.892	0.180	0.002	1.197	1.067–1.343
Age																
6–18	reference				reference				reference				reference			
18–40	−0.536	<0.001	0.585	0.476–0.720	−0.574	<0.001	0.563	0.459–0.691	−0.537	<0.001	0.584	0.469–0.729	−0.509	<0.001	0.601	0.494–0.732
40–60	−1.171	<0.001	0.310	0.241–0.400	−1.163	<0.001	0.312	0.243–0.402	−0.866	<0.001	0.421	0.314–0.564	−0.859	<0.001	0.423	0.328–0.547
>60	−1.653	<0.001	0.191	0.147–0.249	−1.626	<0.001	0.197	0.152–0.255	−1.651	<0.001	0.192	0.134–0.274	−1.783	<0.001	0.168	0.124–0.228
Education level																
low	reference				reference				reference				reference			
medium	0.399	<0.001	1.490	1.306–1.700	0.400	<0.001	1.492	1.308–1.701	0.667	<0.001	1.949	1.648–2.304	0.693	<0.001	2.000	1.728–2.314
high	0.779	<0.001	2.180	1.809–2.626	0.796	<0.001	2.216	1.841–2.668	0.937	<0.001	2.553	2.021–3.225	1.025	<0.001	2.786	2.277–3.410
Occupational status																
health-related industries	0.261	<0.001	1.298	1.127–1.496	0.229	0.001	1.257	1.092–1.446	0.158	0.061	0.848	0.992–1.381	0.096	0.197	1.101	0.951–1.274
others	reference				reference				reference				reference			
Marriage status																
married	reference				reference				reference				reference			
bachelor	0.439	<0.001	1.551	1.266–1.899	0.433	<0.001	1.542	1.262–1.884	0.362	0.001	1.437	1.152–1.792	0.526	<0.001	1.692	1.393–2.055
divorced/widowed	0.300	0.079	1.350	0.965–1.888	0.291	0.088	1.338	0.957–1.871	0.268	0.305	1.307	0.783–2.181	0.345	0.108	1.412	0.927–2.151
Obesity																
no	reference				reference				reference				reference			
yes	0.132	0.132	1.141	0.961–1.354	0.128	0.143	1.136	0.958–1.347	0.175	0.078	1.191	0.981–1.447	0.039	0.666	1.039	0.872–1.238
NCD																
no	reference				reference				reference				reference			
yes	−0.026	0.746	0.975	0.834–1.139	−0.046	0.563	0.955	0.817–1.116	0.031	0.793	1.031	0.820–1.298	0.115	0.244	1.122	0.924–1.362
Weight loss/shaping in the past month																
no	reference				reference				reference				reference			
yes	0.533	<0.001	1.704	1.375–2.111	0.534	<0.001	1.706	1.380–2.110	0.618	<0.001	1.855	1.510–2.278	0.526	<0.001	1.692	1.396–2.051
Mood swing in the past month																
no	reference				reference				reference				reference			
yes	0.273	0.147	1.314	0.908–1.902	0.277	0.139	1.319	0.914–1.904	−0.024	0.908	0.976	0.649–1.469	0.192	0.287	1.212	0.851–1.727

^(1)^ Total beverages; ^(2)^ sugar-sweetened beverages; ^(3)^ non-sugar sweetened beverages; ^(4)^ ready-to-drink beverages; ^(5)^ logistic regression analyses statistics.

## Data Availability

The original contributions presented in this study are included in the article, further inquiries can be directed to the corresponding author.
